# TRIM59 knockdown inhibits cell proliferation by down-regulating the Wnt/β-catenin signaling pathway in neuroblastoma

**DOI:** 10.1042/BSR20181277

**Published:** 2019-01-18

**Authors:** Gang Chen, Weicheng Chen, Ming Ye, Weiqiang Tan, Bing Jia

**Affiliations:** Department of Pediatric Cardiothoracic Surgery, Children’s Hospital of Fudan University, 399 Wanyuan Rd, Shanghai, P.R. China

**Keywords:** β-catenin, neuroblastoma, TRIM59, Wnt

## Abstract

Neuroblastoma is the most common tumor in children, with a very poor prognosis. It is urgent to identify novel biomarkers to treat neuroblastoma, together with surgery, chemotherapy, and radiation. Human tripartite motif 59 (TRIM59), a member of the TRIM family, has been reported to participate in several human tumors. However, the exact role of TRIM59 in neuroblastoma is unknown. In the present study, real-time PCR and Western blot were used to measure mRNA and protein levels of TRIM59 in four neuroblastoma cell lines and in neuroblastoma tissues. Lentiviruses targeting TRIM59 were used to up/down-regulate TRIM59 expression levels. Cell Counting Kit-8 and Annexin-V/PI were used to analyze cell proliferation and apoptosis in neuroblastoma cell lines. Our data showed that TRIM59 knockdown inhibits cell proliferation while inducing apoptosis in SH-SY5Y and SK-N-SH neuroblastoma cell lines. TRIM59 knockdown up-regulated expression of Bax and Bim and down-regulated levels of Survivin, β-catenin, and c-myc. Interestingly, the inhibition of cell proliferation caused by TRIM59 knockdown could be blocked by LiCl, which is an agonist of Wnt/β-catenin signaling pathway. In contrast, TRIM59 overexpression could increase cell proliferation, up-regulate Survivin, β-catenin and c-myc, down-regulate Bax and Bim, and these effects could be blocked by XAV939, which is an inhibitor of Wnt/β-catenin signaling pathway. In addition, TRIM59 was up-regulated and positively related with β-catenin in neuroblastoma tissues. In conclusion, TRIM59 was up-regulated in neuroblastoma, and TRIM59 knockdown inhibited cell proliferation by down-regulating the Wnt/β-catenin signaling pathway in neuroblastoma.

## Introduction

Neuroblastoma is the most common extracranial solid tumor in children up to 5 years old, accounting for nearly 15% of pediatric cancer deaths [1]. Heterogeneity is a hallmark of neuroblastoma, spontaneous regression can occur in some metastatic cases, and fatal tumor progression can also happen in well-treated cases [2]. According to the International Neuroblastoma Staging System, which integrates age, stage, and pathological features, patients are categorized into different (i.e., low, mediate, or high) risk groups [3]. Based on current treatments, including surgery, chemotherapy and radiation, high-risk neuroblastoma patients have a very poor prognosis. Five-year survival is only 20% for this malignancy [4]. Hence, more effective therapies and novel biomarkers are needed.

The Wnt/β-catenin signaling pathway is a vital pathway involved in various cancers, including neuroblastoma [5]. The pathway is highly conserved and regulates a large number of biological processes, such as cell proliferation, cell polarity, apoptosis, and cell differentiation [6]. Aberrant regulation of Wnt/β-catenin signaling is commonly found in human tumors [7–9]. Wnt/β-catenin signaling has been defined by two decades of previous studies. In the absence of Wnt, the Axin complex, which is composed of Axin (the scaffolding protein), adenomatous polyposis coli gene product (APC, the tumor suppressor), glycogen synthase kinase 3 (GSK3), and casein kinase 1 (CK1), degrades β-catenin in the cytoplasm. In the presence of Wnt, the degradation of β-catenin, mediated by the Axin complex, is inhibited, and β-catenin enters the nucleus to combine with T-cell factor/lymphoid enhancer factor, thereby activating expression of target genes [10]. In neuroblastoma, Wnt/β-catenin signaling is inhibited by WIF-1, which is a putative tumor suppressor [11]. Inhibition of TNKS1 decreases the viability of neuroblastoma cell lines via blocking Wnt/β-catenin signaling [12].

The human tripartite motif (TRIM) family is composed of more than 77 members that play roles in many biological processes, such as cell proliferation, apoptosis, development, differentiation, and oncogenesis [13–16]. Most members of the TRIM family contain a highly conserved RBCC domain, which is composed of RING (really interesting new gene), one or two B-box motifs, and a coiled-coil region [17]. TRIM59, a member of the TRIM family, has been reported to participate in several type of human tumor. TRIM59 was first classified as an oncogene in 2011 [18]. Further, TRIM59 promotes cell proliferation in prostate cancer via the regulation of cell cycle progression [19]. Oncogenic abilities of TRIM59 have been observed in osteosarcoma [20]. In addition, knockdown of TRIM59 inhibits cervical cell proliferation and migration [21]. Therefore, TRIM59 may be considered a novel multiple tumor marker for early detection [20]. However, the exact role of TRIM59 in neuroblastoma still needs to be delineated.

In the present study, we identified the function of TRIM59 in neuroblastoma for the first time, suggesting that TRIM59 may act as oncogene via the regulation of Wnt/β-catenin signaling.

## Materials and methods

### Samples and cell culture

Tumor samples and matched adjacent noncancerous samples from 30 patients with neuroblastoma were collected, with the patients’ written consent and the agreement of the Children’s Hospital of Fudan University.

Human neuroblastoma SH-SY5Y, SK-N-SH, and NB-1 cell lines were obtained from the Cell Bank of the Chinese Academy of Sciences (Shanghai, China). The SK-N-BE2 cell line was obtained from American Type Culture Collection (ATCC, Manassas, VA, U.S.A.).

SH-SY5Y cells were cultured in MEM-F12 medium (Invitrogen, Carlsbad, CA, U.S.A.) containing 10% fetal bovine serum (FBS) and 1% NEAA (Invitrogen, Carlsbad, CA, U.S.A.). SK-N-SH cells were cultured in MEM medium (Invitrogen, Carlsbad, CA, U.S.A.) containing 10% FBS and 1% NEAA. SK-N-BE2 cells were cultured in MEM-F12 medium containing 10% FBS and 1% NEAA. NB-1 cells were cultured in MEM medium containing 10% FBS and 1% NEAA. All cells were cultured under an atmosphere of 5% CO_2_ at 37°C.

### Real-time PCR

TRIzol Reagent (Invitrogen, Carlsbad, CA, U.S.A.) was used to extract total RNA of samples and cell lines. cDNA synthesis was performed using the Reverse Transcription System (Promega, Madison, WI, U.S.A.). SYBR Green qPCR Mix (Thermo Fisher Scientific Inc, Grand Island, NY, U.S.A.) was used to perform real-time PCR. Reactions were performed on an ABI 7300 system (Applied Biosystems, Foster City, CA, U.S.A.). The relative expression of target genes was normalized to GAPDH and calculated using the 2^−ΔΔ*C*^_t_ method. All real-time PCR reactions were performed in triplicate. Primers used were as follows:

**Table:** 

TRIM59:	Primer F	5′ TGCCTTACCATAGGTCAAC 3′
	Primer R	5′ GATTGCCAACATCACAGAG 3′
β-Catenin:	Primer F	5′ CCTCCAGGTGACAGCAATCAG 3′
	Primer R	5′ GCCCTCTCAGCAACTCTACAG 3′
GAPDH:	Primer F	5′ AATCCCATCACCATCTTC 3′
	Primer R	5′ AGGCTGTTGTCATACTTC 3′

### Western blot

Samples or cells were harvested using RIPA lysis buffer (Solarbio, Beijing, China), washed twice with cold PBS, and collected by centrifugation at 12000×***g*** for 10 min at 4°C. Bicinchoninic acid (BCA) protein assay kit (Thermo Fisher Scientific Inc, Grand Island, NY, U.S.A.) was used to measure protein concentration. An equal amount (20 μg) of cell lysates was used to perform SDS/PAGE. Anti-β-catenin and GAPDH antibodies for Western blotting were purchased from Cell Signaling Technology. All other antibodies, including TRIM59, Survivin, Bim, c-myc, and Bax, were obtained from Abcam.

### Lentivirus

TRIM59 knockdown and overexpression lentiviruses were synthesized by the Genechem Company (Shanghai, China). Three knockdown lentiviruses were synthesized to ensure knockdown efficiency, two of which were chosen to complete the research for their superior knockdown effect. The sequences used were as follows:

shTRIM59-1: 5′-GGAAGCTGTTCTCCAGTAT-3′;
shTRIM59-2: 5′-GAAGAGTCTCCACTTAAAT-3′;
shTRIM59-3: 5′-GAATGGAGCAGAACAGAAA-3′;

Cells were plated into six-well plates and cultured overnight. TRIM59 knockdown or overexpression lentivirus was added into the wells, according to lentivirus colony forming unit (CFU). After a 48-h transduction, part of the cells was used to measure infection efficiency by real-time PCR and Western blot, while the remaining cells were continued in culture for other experiments.

### Cell proliferation and apoptosis

Cell proliferation was analyzed by a Cell Counting Kit-8 (CCK-8, Beyotime, Shanghai, China). The same number of transduced cells was seeded into 96-well plates and treated with 10 μmol/l XAV939 (Aladdin, China), an inhibitor of Wnt/β-catenin, or 10 mmol/l LiCI (Aladdin, China), which is an agonist of Wnt/β-catenin, followed by culture for 24, 48, and 72 h. Then, the medium of each well was replaced with 100 μl FBS-free medium, including 10 μl CCK-8 and cultured for 1 h. Cell proliferation was assessed by measuring absorbance at 450 nm on microplate reader (Bio-Rad).

Apoptosis was analyzed using an Annexin V Apoptosis Detection Kit (BD Biosciences, San Jose, CA, U.S.A.). The same number of transduced cells was seeded into six-well plates and treated with 10 μmol/l XAV939 or 10 mmol/l LiCI. After culture for various time points, cells were stained with Annexin-V, followed by incubation with PI according to the instructions. Apoptosis was assessed by collecting cells for flow cytometry (BD Biosciences, San Jose, CA, U.S.A.).

### Statistical analysis

All experiments were completed at least three times. Data are shown as the mean ± SD (standard deviation). The statistical analysis of groups was performed using one-way analysis of variance (ANOVA) and the Student’s *t*-test. A *P*-value of <0.05 was considered statistically significant.

## Results

### TRIM59 knockdown inhibits neuroblastoma cell proliferation

**Figure 1:**
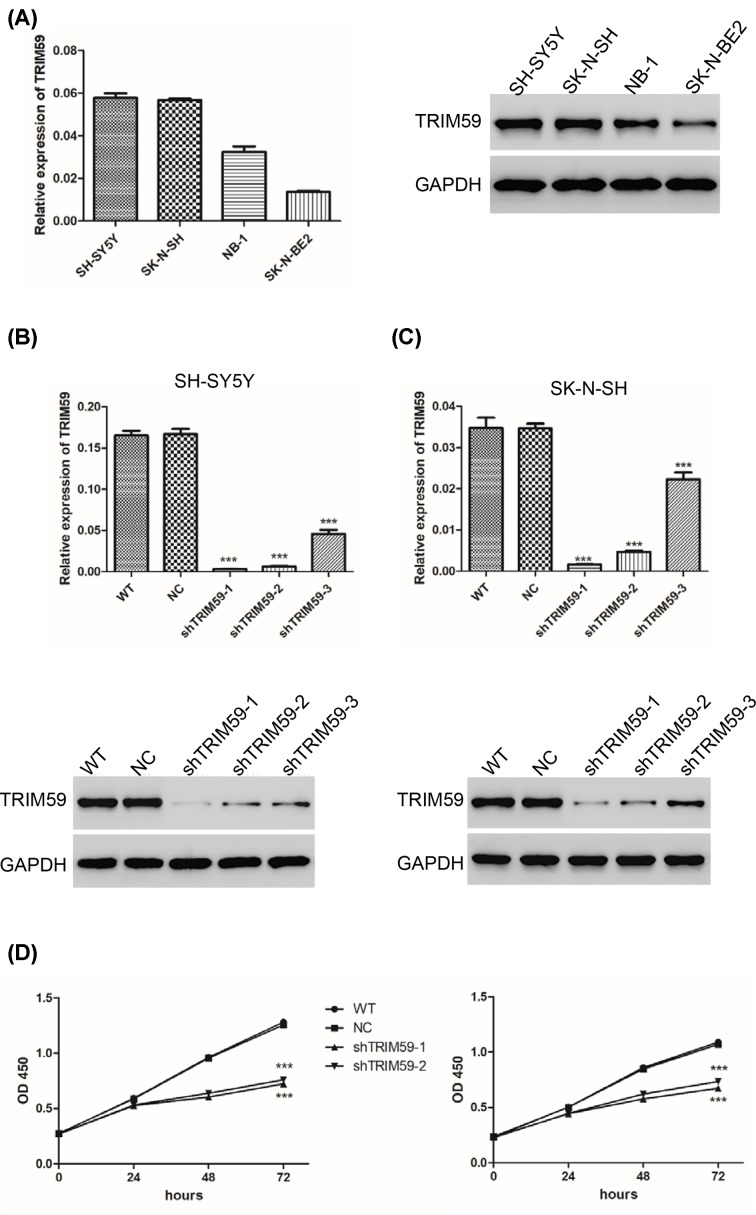
TRIM59 knockdown inhibits neuroblastoma cell proliferation (**A**) Expression levels of TRIM59 were measured by real-time PCR (left panel) and Western blot (right panel) in SH-SY5Y, SK-N-SH, SK-N-BE2, and NB-1 cell lines. (**B** and** C**) Expression levels of TRIM59 were measured after treatment with shTRIM59 lentivirus in SH-SY5Y and SK-N-SH cells. (**D**) CCK-8 was used to measure cell proliferation in SH-SY5Y (left panel) and SK-N-SH (right panel) cells. WT: wide type cells; NC: cells transfected with shNC lentivirus; shTRIM59-1: cells transfected with shTRIM59-1 lentivirus; shTRIM59-2: cells transfected with shTRIM59-2 lentivirus (***P<0.001 vs NC).

To explore the function of TRIM59 in neuroblastoma, we first measured expression of TRIM59 in four neuroblastoma cell lines, SH-SY5Y, SK-N-SH, SK-N-BE2, and NB-1. As shown in Figure 1A, we found that TRIM59 was differentially expressed in all four cell lines. Higher levels were found in SH-SY5Y and SK-N-SH cells, and lower levels were observed in the SK-N-BE2 cell line compared with NB-1 cells at both the mRNA and protein level. We next altered the expression levels of TRIM59 using lentivirus. Three shRNAs targeting TRIM59 were used to knockdown TRIM59 in SH-SY5Y and SK-N-SH cell lines. All three shRNAs silenced TRIM59 expression in both cell lines. ShTRIM59-1 and shTRIM59-2 were chosen for further assay as they exhibited superior knockdown efficiency, both at the mRNA and protein levels (Figure 1B,C). After that, cell proliferation was measured. Both SH-SY5Y and SK-N-SH cells exhibited reduced cell proliferation after treatment with shTRIM59 lentivirus (Figure 1D). These results suggest that TRIM59 knockdown inhibits neuroblastoma cell proliferation.

### TRIM59 knockdown induces apoptosis and regulates apoptosis-related proteins in neuroblastoma cells

**Figure 2:**
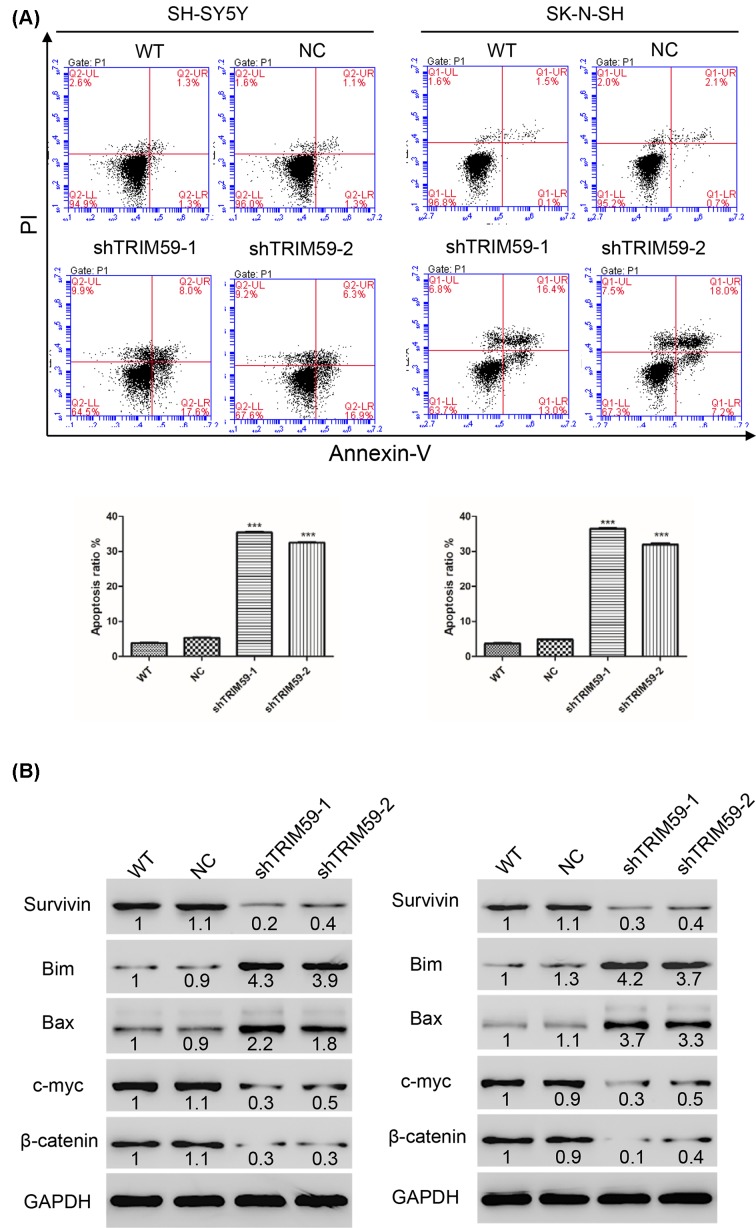
TRIM59 knockdown induces apoptosis and regulates apoptosis-related proteins in neuroblastoma cells (**A**) TRIM59 knockdown induces apoptosis in SH-SY5Y (left panel) and SK-N-SH (right panel) cells. (**B**) Western blot was used to detect expression of some apoptosis-related proteins, including Bax, Bim, Survivin, β-catenin, and c-myc. WT: wide type cells; NC: cells transfected with shNC lentivirus; shTRIM59-1: cells transfected with shTRIM59-1 lentivirus; shTRIM59-2: cells transfected with shTRIM59-2 lentivirus (***P<0.001 vs NC).

Apoptosis plays a key role in tumors. An Annexin-V/PI kit was used to detect apoptosis in neuroblastoma cells transfected with shTRIM59 and NC lentiviruses. Our results showed that cells transfected with shTRIM59 exhibited increased apoptosis rates compared with NC in both SH-SY5Y and SK-N-SH cells (Figure 2A).

Given that TRIM59 knockdown induced apoptosis, we examined expression of apoptosis-related proteins in neuroblastoma cells. TRIM59 knockdown increased expression of Bax and Bim, a pro-apoptotic member of the Bcl-2 family of proteins (Figure 2B). In contrast, TRIM59 knockdown decreased levels of Survivin, which is a member of the inhibitor of apoptosis (IAP) protein family (Figure 2B). It has been reported that Wnt signaling plays a key role in neuroblastoma. After transfection with shTRIM59, we noted that expression of β-catenin, as well as c-myc, was reduced in both SH-SY5Y and SK-N-SH cell lines (Figure 2B).

### TRIM59 regulates cell proliferation and apoptosis through Wnt/β-catenin signaling

**Figure 3:**
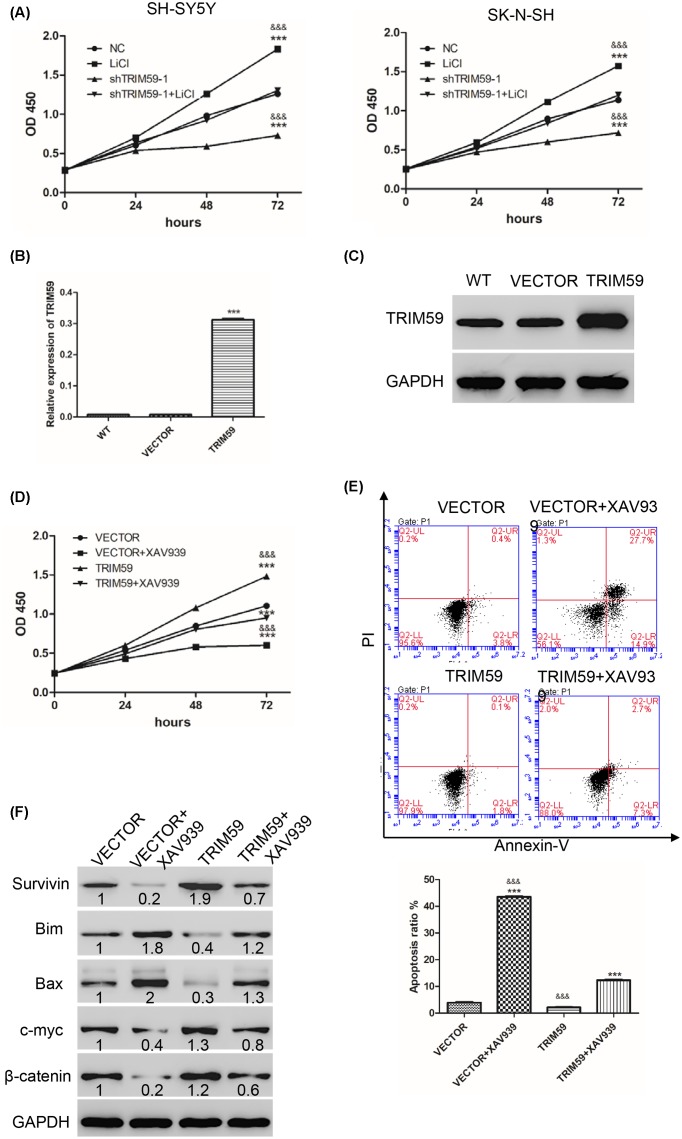
TRIM59 regulates cell proliferation and apoptosis by Wnt/β-catenin signaling (**A**) CCK-8 was used to measure cell proliferation after treatment with LiCl or/and shTRIM59 in SH-SY5Y (left panel) and SK-N-SH (right panel) cells. WT: wide type cells; LiCl: cells treated with 10 mmol/l LiCI; shTRIM59-1: cells transfected with shTRIM59-1 lentivirus; shTRIM59-1+ LiCI: cells treated with 10 mmol/l LiCI and transfected with shTRIM59-1 lentivirus (***P<0.001 vs NC; ^&&&^*P*<0.001 vs shTRIM59-1+ LiCI). (**B** and **C**) Expression of TRIM59 was measured by real-time PCR and Western blot. (**D**) CCK-8 was used to measure cell proliferation. (**E**) Annexin-V/PI kit was used to detect apoptosis. (**F**) Western blot was used to detect expression of some apoptosis-related proteins, including Bax, Bim, Survivin, β-catenin, and c-myc. VECTOR: cells transfected with VECTOR lentivirus; VECTOR+XAV939: cells transfected with VECTOR lentivirus and treated with 10 μmol/l XAV939; TRIM59: cells treated with TRIM59 lentivirus; TRIM59+XAV939: cells treated with TRIM59 lentivirus and treated with 10 μmol/l XAV939 (***P<0.001 vs VECTOR; ^&&&^*P*<0.001 vs TRIM59+ XAV939).

According to our results (Figures 1D and 2B), TRIM59 knockdown inhibited both cell proliferation and expression of β-catenin. We hypothesized that the effects caused by TRIM59 knockdown may occur via the Wnt/β-catenin signaling pathway. First, cells were treated with LiCl, an agonist of Wnt/β-catenin or/and shTRIM59. Then, CCK-8 was used to measure cell proliferation in neuroblastoma cells. As shown in Figure 3A, compared with NC, shTRIM59 inhibited cell proliferation, while LiCl promoted cell proliferation. Cells treated with both shTRIM59 and LiCl showed significantly higher cell proliferation than shTRIM59 but lower than LiCl, with no differences compared with the NC group in either SH-SY5Y or SK-N-SH cells. These results indicate that the inhibition of cell proliferation caused by TRIM59 knockdown is blocked by LiCl.

Further, we used TRIM59 lentivirus together with XAV939, an inhibitor of Wnt/β-catenin, to confirm our hypothesis. TRIM59 lentivirus significantly increased mRNA and protein levels of TRIM59 in SK-N-BE2 cells (Figure 3B,C). Next, cell proliferation and apoptosis were measured. As shown in Figure 3D,E, compared with VECTOR, the XAV939 group significantly inhibited cell proliferation, while promoting apoptosis. The TRIM59 group significantly promoted cell proliferation but inhibited apoptosis. Cells treated with both TRIM59 and XAV939 showed significantly higher/lower cell proliferation/apoptosis than XAV939 but lower/ higher than TRIM59, respectively. Furthermore, after treatment with XAV939, protein levels of Surivivin, c-myc, and β-catenin were decreased, while protein levels of Bim and Bax were increased compared with VECTOR. However, cells treated with TRIM59 exhibited the opposite results. Cells treated with both TRIM59 and XAV939 showed significantly higher levels of Surivivin, c-myc, and β-catenin than XAV939 but lower than TRIM59. Nevertheless, cells treated with both TRIM59 and XAV939 showed significantly lower levels of Bim and Bax than XAV939 but higher than TRIM59 (Figure 3F). Therefore, TRIM59 increases cell proliferation and inhibits apoptosis via activation of the Wnt/β-catenin signaling pathway.

### TRIM59 is up-regulated and positively related with β-catenin in neuroblastoma tissues

**Figure 4:**
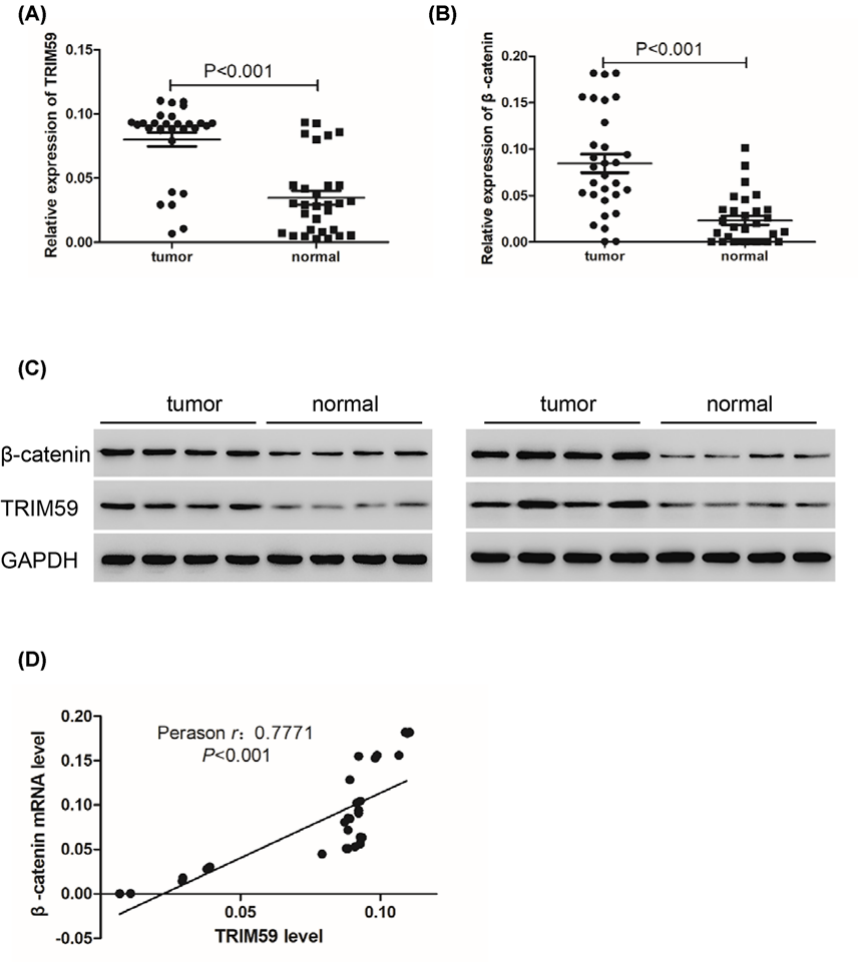
TRIM59 is up-regulated and positively correlated with β-catenin in neuroblastoma tissues (**A**) Real-time PCR was used to detect expression levels of TRIM59 in neuroblastoma tissues and normal tissues (*n*=30). (**B**) Real-time PCR was used to detect expression levels of β-catenin in neuroblastoma tissues and normal tissues (*n*=30). (**C**) Western blot was used to detect expression levels of TRIM59 and β-catenin in neuroblastoma tissues and normal tissues (*n*=8). (**D**) Pearson correlation analysis demonstrated a positive correlation between TRIM59 and β-catenin in neuroblastoma tissues (*n*=30).

Given that TRIM59 knockdown inhibited cell proliferation, we next measured expression levels of TRIM59 in human neuroblastoma tissues. As shown in Figure 4A,C, TRIM59 was highly overexpressed in neuroblastoma tissues compared with normal tissues due to TRIM59 regulating cell proliferation and apoptosis through Wnt/β-catenin signaling. We further detected expression levels of β-catenin, revealing that β-catenin is also highly overexpressed in neuroblastoma tissues compared with normal tissues (Figure 4B,C). In addition, Pearson correlation analysis demonstrated a positive correlation between TRIM59 and β-catenin in neuroblastoma tissues (*r*  = 0.7771) (Figure 4D).

## Discussion

Neuroblastoma is the most common malignant tumor. High-risk neuroblastoma patients have very low cure rates, despite therapy comprises combined treatments, including surgery, chemotherapy, and radiation [22]. Clearly, understanding the pathogenesis of neuroblastoma would facilitate identification of novel biomarkers to exploit more effective therapies.

The TRIM family contains the highly conserved RBCC domain and participates in many biological processes [17]. TRIM59, a member of the TRIM family, has been reported to play a role in several types of human tumor. In the present study, we first assessed the function of TRIM59 in neuroblastoma. Our results demonstrated that both mRNA and protein levels of TRIM59 were overexpressed in human neuroblastoma tissues (Figure 4). In *in vitro* experiments, expression of TRIM59 was down/up-regulated by employing TRIM59 targeting lentivirus, and we observed that TRIM59 knockdown inhibited cell proliferation and promoted cell apoptosis in SH-SY5Y and SK-N-SH cells (Figures 1D and 2A). In contrast, TRIM59 overexpression promoted cell proliferation in SK-N-BE2 cells (Figure 3D), suggesting that TRIM59 may play an oncogenic role in neuroblastoma. These results are consistent with some previous studies [18–21].

We further characterized TRIM59-mediated regulation of some apoptosis-related proteins. Silencing of TRIM59 increased the expression of Bax and Bim but decreased the amounts of Survivin (Figure 2B). Bax and Bim are members of Bcl-2 family that play key roles in the regulation of apoptosis. Survivin, a member of the IAP family, plays a key role in cell proliferation and cell survival via inhibition of apoptosis [23]. However, overexpression of TRIM59 exhibited the opposite effects (Figure 3F). Next, we demonstrated that TRIM59 also regulates β-catenin, a key member of Wnt/β-catenin signaling. The silencing of TRIM59 reduced β-catenin in SH-SY5Y and SK-N-SH cell lines (Figure 2B), indicating that the inhibition of TRIM59 leaded to degradation of β-catenin. We also found that c-myc, a downstream target protein of β-catenin, was down-regulated, demonstrating that Wnt/β-catenin signaling was inhibited. In contrast, overexpression of TRIM59 increased β-catenin in SK-N-BE2 cells, as well as c-myc (Figure 3F).

To further confirm our findings, LiCl and XAV939, an agonist and inhibitor of Wnt/β-catenin, respectively, together with TRIM59 lentivirus, were used to treat cells. The results showed that LiCl blocked the decrease of cell proliferation in response to TRIM59 inhibition (Figure 3A). Moreover, XAV939 blocked all effects caused by TRIM59 overexpression (Figure 3). In addition, β-catenin was up-regulated and was positively correlated with TRIM59 in neuroblastoma tissues.

Therefore, TRIM59 regulates cell proliferation and apoptosis via Wnt/β-catenin signaling. However, these results still need to be demonstrated *in vivo*.

In summary, we demonstrated that knockdown TRIM59 inhibits cell proliferation and induces apoptosis in neuroblastoma cells. Furthermore, one mechanism whereby this occurs seems to be through the inhibition of Wnt/β-catenin signaling.

## Abbreviations

APC: adenomatous polyposis coli
CCK-8: Cell Counting Kit-8
CK1: casein kinase 1
GSK3: glycogen synthase kinase 3
IAP: inhibitor of apoptosis
NEAA: non-essential amino acids
TNKS1: tankyrase 1
TRIM: tripartite motif
